# Assessment of relationship of mental health and quality of life of COVID-19 Survivors in selected hospitals of Tehran University of Medical Sciences, 2021

**DOI:** 10.1192/j.eurpsy.2023.772

**Published:** 2023-07-19

**Authors:** H. Khosravi, L. Sayadi, E. Mohammadnejad

**Affiliations:** 1 Nursing and midwifery, semnan university of medical sciences, semnan; 2 Nursing and midwifery, tehran university of medical sciences, tehran, Iran, Islamic Republic Of

## Abstract

**Introduction:**

Survivors from Covid-19 are more prone to psychological distress due to their experience of illness, hospitalization, and severe conditions. Diseases can also affect patients’ quality of life. Nursing of these patients is not limited to hospitalized patients and paying attention to the mental health status and quality of life of patients is one of the roles and responsibilities of nurses. The aim of this study was to determine the relationship between mental health and quality of life of Covid-19 survivors in selected hospitals of Tehran University of Medical Sciences in 2021.

**Objectives:**

Due to the disturbance in mental health and the decrease in the quality of life of the survivors of this disease, the results of the research can be used for policy making and rehabilitation planning for those who have recovered from this disease. Also, considering that the covid-19 virus may remain for years and infect many people, supporting and controlling the physical and mental conditions of these people in the long term can help to increase the quality of life and improve their mental health.

**Methods:**

This study is a cross-sectional study in which the mental health and quality of life of 276 survivors of Covid-19 who were hospitalized in three hospitals of Imam khomeini, Shariati and Baharloo between February 2020 and July 2020 were examined. Data were collected by using demographic and clinical information questionnaire, 12-item general health questionnaire and 36-item quality of life questionnaire. Data analysis was performed using SPSS16 software at a significance level of 0.05.

**Results:**

The results showed that the general health (mental health) score of the participants in this study one year after Covid-19 was equal to 6/26 ± 2/75 and 231 survivor of Covid-19 (83.7%) score more than 3 they had. Regarding quality of life, the results showed that the dimensions of energy and vitality, and general health, had the lowest scores with an average of 53.3 and 54.71 (out of 100 points), respectively.There is an inverse relationship between mental health and all aspects of quality of life, so that with the deterioration of mental health status in Covid-19 survivors, their quality of life score decreases.

**Conclusions:**

The results of the study showed that the mental health status of a large percentage of Covid-19 survivors is impaired and there is also a significant and inverse relationship between the total score of mental health and dimensions of quality of life and their total score of quality of life. Paying attention to the mental health problems of Covid-19 survivors by health system staff, especially nurses, and choosing supportive strategies for them is one of the priorities that should be considered. Psychological support for these people can improve their mental health and indirectly increase their quality of life.

**Disclosure of Interest:**

None Declared

